# The Effect of Liquid Slurry-Enhanced Corrosion on the Phase Composition of Selected Portland Cement Pastes

**DOI:** 10.3390/ma14071707

**Published:** 2021-03-30

**Authors:** Karol Durczak, Michał Pyzalski, Krzysztof Pilarski, Tomasz Brylewski, Agnieszka Sujak

**Affiliations:** 1 Department of Biosystems Engineering, Faculty of Environmental and Mechanical Engineering, Poznań University of Life Sciences, Wojska Polskiego 50 Street, 60-627 Poznań, Poland; karol.durczak@up.poznan.pl (K.D.); krzysztof.pilarski@up.poznan.pl (K.P.); agnieszka.sujak@up.poznan.pl (A.S.); 2 Faculty of Materials Science and Ceramics, AGH University of Science and Technology, A. Mickiewicza 30 Street, 30-059 Kraków, Poland; brylew@agh.edu.pl

**Keywords:** portland cement, biocorrosion, microstructure, phase and chemical composition, taumasite

## Abstract

This paper presents the scientific problem of the biological corrosion of Portland cements and its effects on the phase composition of cement pastes after the corrosion process in the environment of reactive media from the agricultural industry. Seven Portland cements produced from different cement plants exposed to pig slurry and water as a reference medium for a period of six weeks were tested. After the exposure process in both of the above-mentioned reaction environments, the hydrating cement pastes were characterized in terms of their phase composition using the XRD method and were also subjected to morphological observations and a chemical composition analysis with the application of SEM and EDS methods. The results of these studies indicate the presence of a biological corrosion product in the form of taumasite [C_3_S·CO_2_·SO_3_·15H_2_O], which is a phase formed as a result of the reaction of dead matter (cement paste) with living matter, caused by the presence of bacteria in pig slurry. In addition to taumasite, the tested samples also showed the presence of the hydration product of Portland cements named portlandite (Ca(OH)_2_). Moreover, unreacted phases of cement clinker, i.e., dicalcium silicate (C_2_S) and tricalcium aluminate (C_3_A), were detected. Based on microscopic observations and analyses of the chemical composition of selected areas of the samples, the presence of the taumasite phase and compact areas of pseudo-crystalline C-S-H phases with different morphological structures, derived from the hydration products of cements doped with ions originating from the corrosive environment, were confirmed.

## 1. Introduction

The intensive development of agriculture contributes to the increase in the supply of animal feed materials, requiring appropriate storage conditions. As a result, the production of organic waste that needs disposal has expanded [1,2]. Therefore, the increasing demand for concrete materials has been observed.

During agricultural production, in many cases maize silage, beet pulp, and manure are being stored on concrete surfaces. Moreover, organic waste utilization processes are carried out both under aerobic (composting) and anaerobic conditions, as well as methane fermentation processes in closed concrete tanks [3]. Most commonly, biocorrosion of concretes is observed during the storage of maize silage and in anaerobic waste disposal processes, and when leachate appears while composting. It is, however, related to the appearance of variable environmental conditions at the interface between the stored or utilized material and the concrete surface. This phenomenon is related to the insufficient durability of agricultural constructions. As a result, additional costs related to their repair and maintenance appear.

In recent years, a number of concrete structures used in agriculture have shown severe durability problems. It turns out that many constructions exposed to aggressive environments encounter problems with biocorrosion. This process occurs especially in places such as storage slabs, composting or biogas plants and in other constructions such as sewage, underground and hydraulic structures, chemical plants and industrial structures. Such a situation requires taking actions aimed at minimizing the destructive influence of chemical and organic compounds present in aggressive materials of plant and animal origin on the properties of structural concretes.

Optimal concrete construction mixtures should be searched for in terms of cost and durability. This would allow, already at the stage of designing the installation for storing of agricultural products, for the minimization of the negative effects in the future.

The processes taking place between the surface and the surrounding environment are called corrosion (from Latin *corrosio*–eating away). Corrosion is defined as a single or co-process leading to the gradual degradation of materials. The nature of the biocorrosion is essentially guided by the type of material the damaged element is made of. Corrosion can include several processes of physical (when the degradation of material is the result of physical processes without the participation of chemical reactions), electrochemical (when there is an electrolyte pair–metal or metal alloy, and therefore mainly in aqueous solutions), chemical (usually taking place with the participation of water as a very good solvent) or microbiological character (biocorrosion caused by biotic factors) [4,5]. These processes affect the durability of structural elements, which translates into the clear-cut service life of a building or specific structure.

Biocorrosion is a process initiated by microorganisms. Microbes can facilitate and accelerate this unfavorable phenomenon without changing its electrochemical nature. Microorganisms contribute various effects to the corrosion process resulting from their interaction with the environment surrounding the concrete surface. As their involvement can be intense, the significant damage increase to concrete elements can be expected.

Sulfate ions are commonly responsible for the damage that occurs in concrete elements. They are formed in the processes of the anaerobic decomposition of organic matter, which in developed countries is becoming one of the main methods of utilizing organic materials from agri-food processing. The higher the concentration of sulfate ions, the shorter the time that concrete materials can be destroyed, which leads to the decrease of their structural and mechanic properties. In addition, concretes are reinforced with steel bars, which also corrode under the influence of sulfate ions, which is consequently an extensive threat to construction structures. Sulfates are a medium for SRB (sulfate-reducing bacteria), which transform into highly corrosive sulfides [6].

Chloride ions are another group that act corrosively to concrete elements and appear in the corrosive solution as a result of the mineralization of organic biomass in the processes of its decomposition. They take part in the dissolution of many salts and metals from agricultural objects. Their activity in the aquatic environment is due to their high reactivity. They supersede calcium, which is used in the form of slaked lime in concrete mortars. As a result, the disintegration of molecular bonds between individual components of concrete mortars occurs via crumbling, which in turn leads to the weakening of the structure of building objects [7]. This problem is known mainly in offshore concrete structures [8]. This obstacle may be overcome by the application of the superhydrophobic concrete with anti-corrosion and stable mechanical properties [9].

Microbial corrosion (MIC) is a common problem faced by all sectors of the economy. Despite many studies, the biocorrosion MIC of concrete elements remains the subject of many discussions concerning possibilities of slowing it down (inhibition) [10]. This is mainly due to the lack of standardized testing and evaluation methods. In addition, it is difficult to establish quantitative and qualitative relationships between the corrosion behavior observed on site and that obtained in the laboratory tests [11]. This brings the need for further research to deepen our understanding of the various aspects related to the MIC of concrete elements. A critical review on several aspects of microbiologically induced concrete corrosion was presented by Wu et al. [12]. Four main stages have been considered accounting for the MIC process, i.e., the formation of hydrogen sulfide in waste steam, the radiation and buildup of gaseous hydrogen sulfide, the generation of sulfuric acid, and the deterioration of concrete materials. The authors have reviewed and discussed the fundamentals associated with the main events. Based on site investigations and laboratory studies, several aspects of the MIC phenomena were summarized, including corrosion areas, corrosion rates, and the impact of cement and aggregate types.

Many methods have been developed to detect the corrosion of precast concrete elements. Taheri et al. [13] applied a computed tomography as a non-destructive technique for the testing of sewage pipes. Softening of the concrete surface exposed to sewerage has been reported during normal usage. A solution to this problem could be the addition of nitrites to concrete mixes [14].

The aim of the research was to determine the effect of pig slurry on the physicochemical parameters of concrete pastes.

## 2. Materials and Methods

### 2.1. Preparation of Samples from Ordinary Portland Cement (OPC) Pastes

Samples of seven Portland cements of the same type (OPC) were selected for the experiment. A chemical analysis of the selected cement pastes is presented in Table 1.

“CEM I” Portland cements are usually composed of 95% of cement clinker and 5% of calcium sulfate dihydrate as a setting time regulator. In the presented experiment, “pure” cement pastes were used as additives, other than cement clinker, such as fly ash, blast furnace slag, or geoses, could significantly affect the course of corrosion. Carrying out an experiment on cement pastes made of “CEM I” allowed to determine the resistance (or lack of resistance) of these materials to corrosive exposure conditions. The cement pastes tested in this study are of the same strength class—42.5 (this means that after 28 days of setting, they reach the strength of 42.5 MPa). In addition, the cements have a high initial strength, characterized by the symbol “R” (rapid). The exception is the cement from the “Chełm” cement plant, which has the “N” symbol, indicating that the cement has a normal initial strength. Detailed information on the characteristics of the cement paste used in this experiment has been included in the PN-EN-197-1 standard [15].

The samples were formed as rectangular bars with dimensions of 40 mm × 40 mm × 160 mm. Prior to the experiment, the cement paste samples were set for 24 h in standard molding forms. After demolding, the setting process was continued for another 24 h in a climate chamber providing 100% ambient humidity. The bars from cement pastes prepared in this way were placed in tightly closed containers filled with slurry, which was a medium of biological aggression. Bars kept in water were taken as a control.

### 2.2. Conditions of the Biological Corrosion Process

Pig slurry obtained from the industrial fattening of pigs was used as a medium causing biocorrosion. Water with the parameters outlined in Table 2 was used as a control. The pH of the water was 7.3 ± 0.1.

The exposure time of the samples to the corrosive agent was up to six weeks. The chemical composition of liquid pig slurry is given in Table 3. The pH of the pig slurry was 7.4 ± 1.5.

### 2.3. Characterization Methods

The phase composition of the samples was investigated by X-ray diffraction (XRD) using apparatus consisting of a power supply stabilizing the operation of the PW 1140/00/60 X-ray tube and a vertical goniometer PW 1050/50 (Philips, Eindhoven, The Netherlands). The device included a vertically mounted PHILIPS X-ray tube with a Cu anti-cathode and wavelength Ka = 1.54178 Å; an Ni filter was used. A PW 2216/20 “fine focus” X-ray tube with a power of 1.2 kW was used (the applied lamp power was 1 kW, which corresponded to the operation with the lamp voltage = 40 kV and the cathode filament current = 25 mA). A narrow radiation beam with the properly performed adjustment of the diffractometer settings allowed to improve the accuracy of measurement data.

The morphology of the samples was studied using an ultra-high-resolution scanning electron microscope (FEI, Nova NanoSEM 200, Philips, Eindhoven, The Netherlands) equipped with an electron gun with thermal field emission (FEG-Schottky emitter, Philips, Eindhoven, The Netherlands) at an accelerating voltage of 18 kV.

The elemental composition of the tested samples was determined using the EDAX Genesis XM X-ray energy dispersion analyser (EDS) equipped with a scanning electron microscope.

## 3. Results and Discussion

### 3.1. Sample Mass over the Exposure Time of Six Weeks

The data on the measurement of the mass of the samples at the beginning of the test and after two, four, and six weeks, respectively, are shown in Table 4.

The greatest changes in the mass of the samples during the period of six weeks were shown by samples 1 and 6. Sample 7 showed the smallest changes in mass. In the initial phase of the experiment, samples 1 and 6 showed the highest water absorption. Their masses increased by 4.4% and 2.2%, respectively. In the initial phase, no increase in weight was observed for samples 2 and 5. Weight loss, which can be combined with the partial dissolution of the components, was observed in samples 3 and 4. Interestingly, in the samples where weight gain was initially noticed, a decrease of this parameter was noted after four weeks.

### 3.2. Phase Composition of Cement Pastes (Corrosion and Hydration Products)

**Figure 1:**
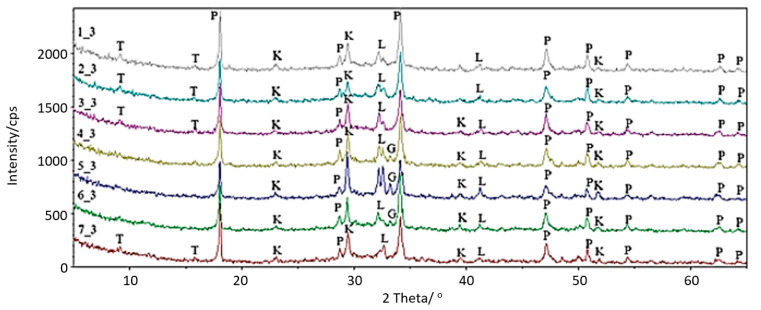
Diffractograms of the examined cement pastes; T-C_3_S·CO_2_·SO_3_·15H_2_O, P-Ca(OH)_2_, K-CaCO_3_, L-C_2_S, G-C_3_A.

The process of the hydration of cement pastes may lead to the formation of the following phases: C-S-H in various forms according to Diamond [16], ettringite, monosulphate, calcium hydroxide (portlandite), hydrated calcium aluminates, and aluminum ferrousates. This, however, strongly depends on the duration of the process. The cement paste samples were exposed to corrosive conditions (thus hydration) in the liquid pig slurry. The phase analysis of cement paste samples subjected to hydration processes under such aggressive exposure conditions is presented in the collective diagram of diffraction patterns (Figure 1) and in Table 5.

The collective analysis of the diffraction patterns of Portland cement pastes did not show any significant differences in the phase composition between the tested samples. It is noteworthy that there are some variable tendencies in the intensities of the main products of hydration or products formed by the secondary reaction of hydrates with the environment. Due to the fact that all samples were made according to the same procedure, and that the same measurement parameters were applied, some tendencies of quantitative changes in the phase composition of cement pastes can be noticed.

The differences in the reflection intensities may result from the variable activity of the cement pastes during hydration or from the formation of higher or smaller amounts of corrosive products of hydration, which may slow down the diffusion processes and then inhibit the growth of subsequent portions of hydration products. The analysis of the diffraction patterns revealed the presence of crystalline phases originating mostly from hydration products of Portland cement. Portlandite [Ca(OH_2_)], which as a result of dynamic carbonation transforms into calcite [CaCO_3_] is one of the most important phases determined in the X-ray pattern. Some amounts of the non-hydrated phase of Portland cement such as dicalcium silicate [C_2_S] and tricalcium aluminate [C_3_A] were also found in the diffraction pattern. Interestingly, taumasite [C_3_S∙CO_2_∙SO_3_∙15H_2_O], which is the only phase that is a product of corrosion and is formed via hydratation process, is present in samples 1_3, 2_3, 3_3, and 7_3.

In cement pastes, taumasite is usually formed at high humidity conditions and at a temperature between 0 and 15 °C, with a simultaneous pH reduction of the Portland cement environment. It can be formed by the direct reaction of the C-S-H phase with carbonates and sulfates. The latter cannot be excluded in the case of the experiment carried out in this work. Another mechanism exists, equally probable in this experiment, of the formation of this dangerous corrosive hydrate through the so-called woodfordite or a solid solution of ettryngite and taumasite. The substrates for the above reaction are the ettrigite phase, calcium carbonate, carbon dioxide, and water. The formation of taumasite by the solid solution is favored by the formation of hydrated calcium aluminates and calcium aluminum ferrates, which makes the formation of this phase more likely in samples where tricalcium aluminate is no longer present. In that case, taumasite peaks are present in the X-ray pattern.

### 3.3. Sample Morphologies and the Chemical Composition of Corrosion Products

In order to identify the biocorrosion process of the samples in the system—cement paste/pig slurry—morphological observations of their transverse fractures were carried out using scanning electron microscopy (SEM). X-ray energy dispersion spectroscopy (EDS) was used to analyse the chemical composition of the corrosion products. For comparative purposes, the results of similar tests were also presented for the system—cement paste/water.

**Figure 2:**
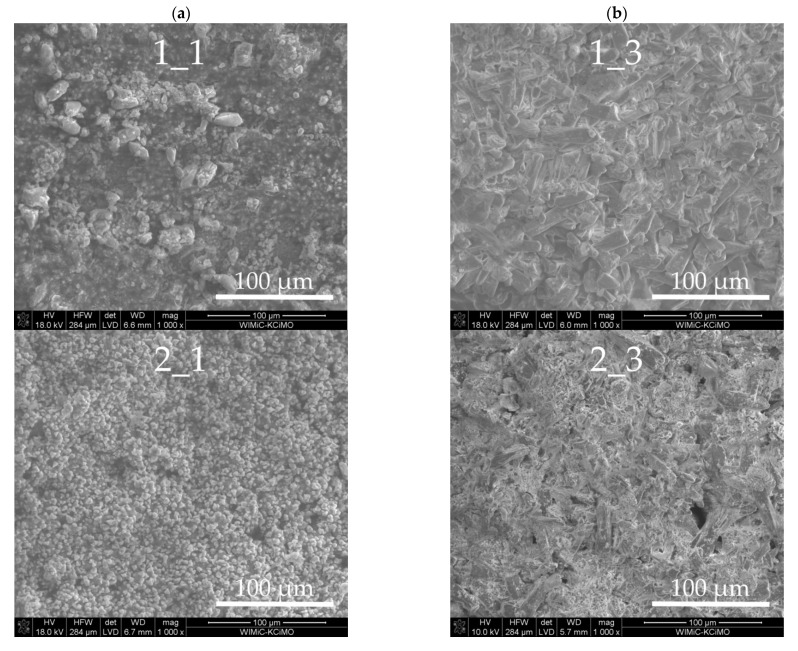

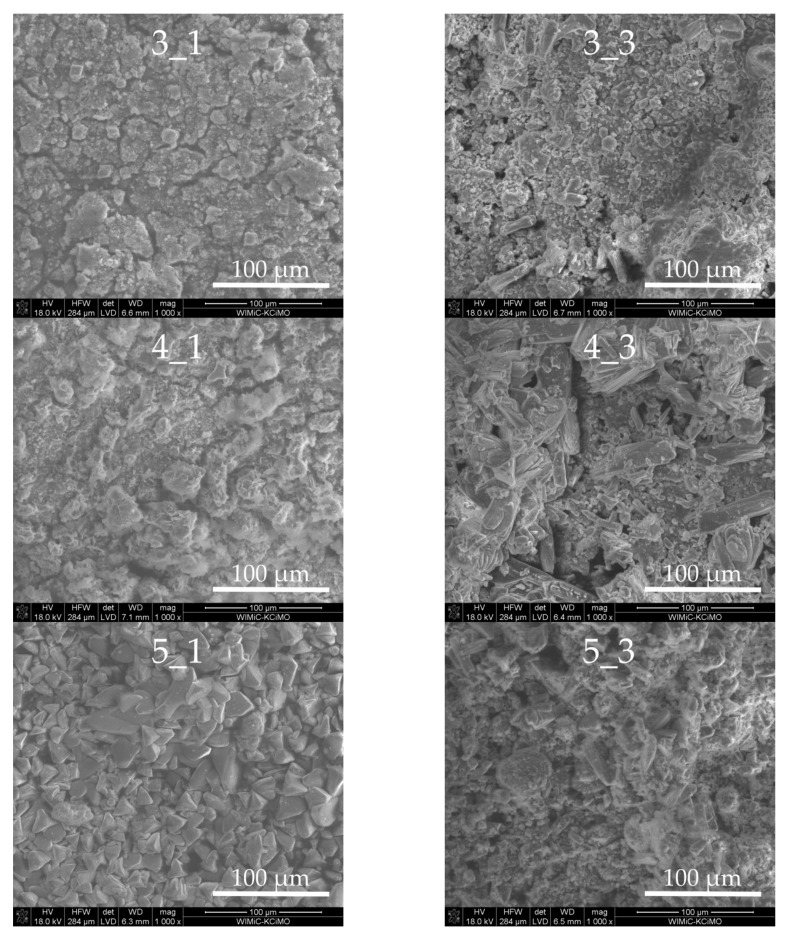

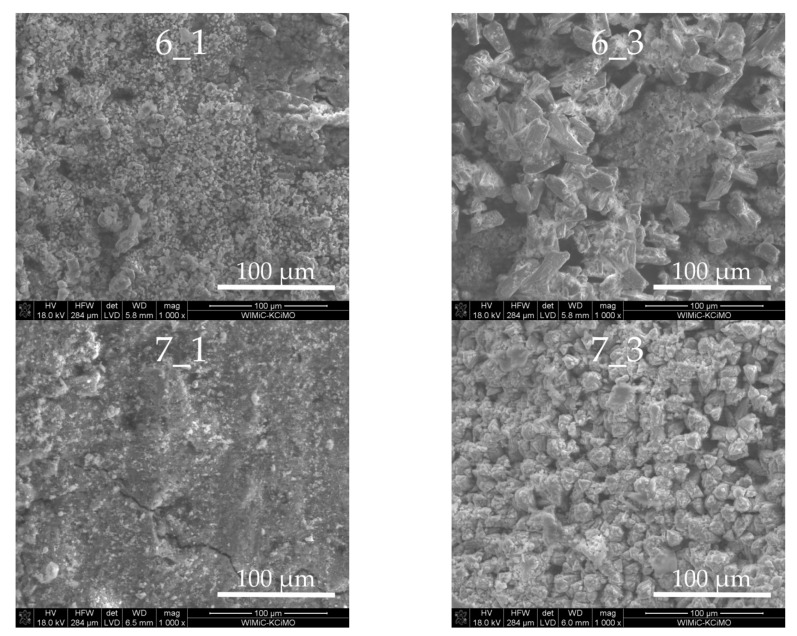
SEM micrographs of the cement pastes obtained after exposure under the conditions of: (**a**) water (samples 1_1–7_1) and (**b**) pig slurry (samples 1_3–7_3).

In Figure 2, panels 1–7 show SEM photomicrographs of the fracture of the cement paste samples exposed in both reaction environments.

**Figure 3:**
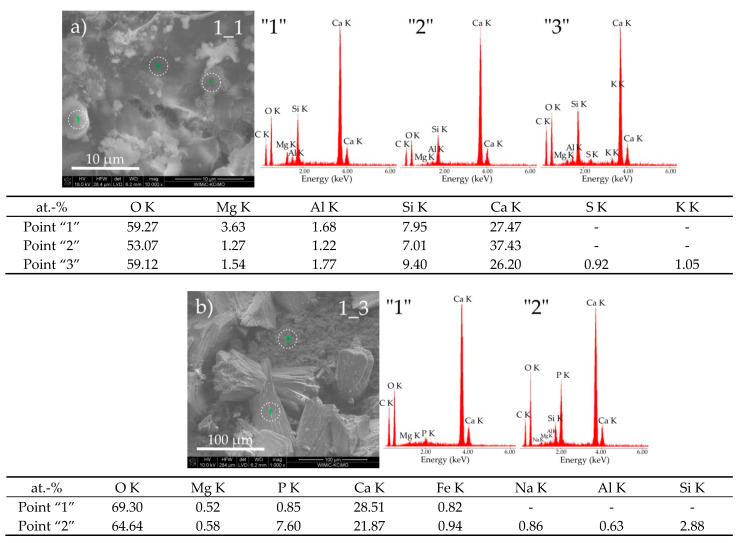
SEM micrographs of cement paste 1 obtained after exposure under the conditions of (**a**) water (sample 1_1) and (**b**) pig slurry (sample 1_3) with EDS quantitative point analyses from areas 1, 2, and 3.

The microscopic observations of the tested samples show a diversified morphological structure depending on the mineral composition of Portland cement and the type of corrosive medium used. In all cases, changes in the microstructure, such as the shape and size of grains and the distribution of phases present in cement pastes, are observed. There are also apparent pits (pores) in the pastes after the corrosion process in the environment of the pig slurry, most clearly observable in samples 4_3 and 6_3. The analysis of the morphological structure of cement paste 1 after its exposure to both reaction media shows a clear change in the shapes and organization of grains, which, in the case of sample 1_3, significantly elongated, reaching sizes from 5 μm to 30 μm (Figure 2, sample 1_3). In turn, sample 1_1 is fine-grained and consists of grains in the form of agglomerates sized between 2 and 20 μm. Both samples are characterized by a compact structure. The results of the chemical composition of their selected areas by the EDS point technique presented in Figure 3a,b indicate significant differences in their chemical composition.

In the case of sample 1_1, the hydration product of alite and belite is visible in the form of the C-S-H phase (point “1”—Figure 3a) with a “honeycomb” mesh structure which, according to classification proposed by Diamond [16], de facto corresponds to the morphological phase II type of the C-S-H. In some areas of the sample, a small amount of sulfur and aluminum was identified (point “3”—Figure 3a), which may indicate the presence of amorphous monosulfate, forming a nanometric mixture with the C-S-H phase.

In the case of sample 1_3 exposed to pig slurry, a different morphological structure is seen compared to the sample kept in water. A considerable phosphorus concentration (above 8% by weight) is noteworthy, especially in the areas of the fine-grained matrix (point “2”—Figure 3b), which are supposed to be loosely bound isomorphic crystals of type III of hydrated calcium silicates. The existence of phosphorus in the sample can be explained by the fact that it is one of the main components of pig slurry (Table 3). Taking into account the shape of the plates and its chemical composition (point “1”—Figure 3b), it could be assumed that they represent portlandite grains.

**Figure 4:**
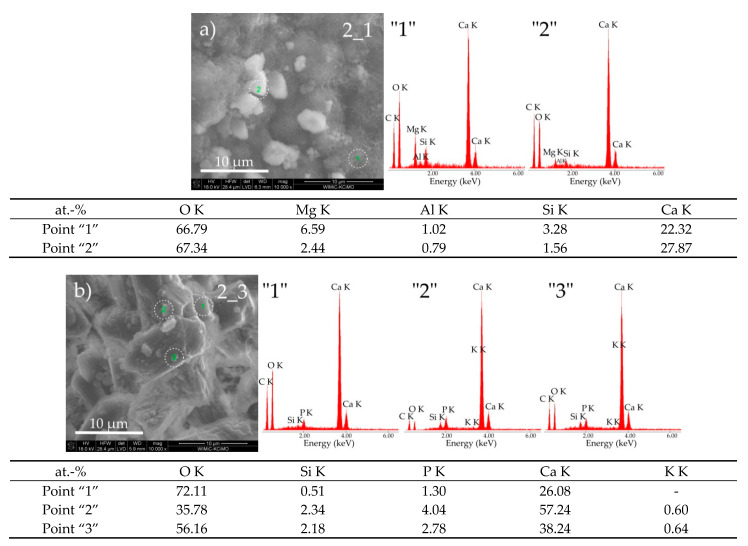
SEM micrographs of cement paste 2 obtained after exposure under the conditions of (**a**) water (sample 2_1) and (**b**) pig slurry (sample 2_3) with EDS quantitative point analyses from areas 1, 2, and 3.

Exposure of cement paste 2 in both reaction environments also leads to noticeable changes in their morphological structure (Figure 2). These differences can most clearly be seen on SEM micrographs at a high magnification (10,000×), as shown in Figure 4a,b, and are also confirmed by the EDS spectra representing the chemical composition taken from selected areas of the tested samples.

Cement paste kept in water consists mainly of agglomerates of portlandite grains with sizes ranging from 1 to 5 μm (point “2”—Figure 4a) surrounded by the mesh-like CSH phase II–honeycomb (point “1”—Figure 4a). The cement paste subjected to biocorrosion consists of grains in the form of oblong agglomerates with random orientation. Their chemical composition approximately corresponds to the nominal composition of the calcium hydroxide phase–portlandite (points “1”, “2” and “3”—Figure 4b). High-intensity peaks in the EDS spectrum may indicate the presence of fine crystalline calcium carbonate with undetermined polymorphism, which will certainly participate in the process of taumasite formation in the reaction with the C-S-H phase. Large pores are visible between these agglomerates. A small concentration of phosphorus in the analysed sample, not exceeding 4% by weight, is noteworthy.

**Figure 5:**
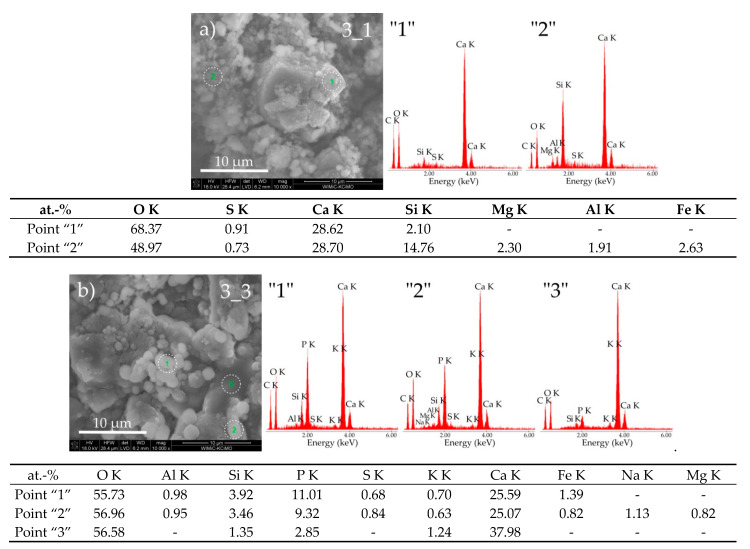
SEM micrographs of cement paste 3 obtained after exposure under the conditions of (**a**) water (sample 3_1) and (**b**) pig slurry (sample 3_3) with EDS quantitative point analyses from areas 1, 2, and 3.

Based on the analysis of the microstructure of sample 3 (Figure 2), it can be concluded that the pig slurry has a slight influence on its corrosion process. Figure 5a,b show the results of EDS analyses of the chemical composition of selected areas of both tested samples together with their SEM micrographs at high magnification (10,000×).

The large grains visible in the SEM photomicrograph probably represent portlandite plates (point “1”—Figure 5a) fused with type III hydrated calcium silicates (point “2”—Figure 5a); amorphous precipitates of monosulfate with a layered structure can be found. In the sample exposed to pig slurry, approximately spherical or oval precipitates can be observed, consisting of secondary crystallized calcium carbonate, which is most likely formed as a result of a topochemical reaction between reactive calcium hydroxide and bacterial waste products such as carbon dioxide (points “1” and “2”—Figure 5b). In the discussed sample, the presence of large grains of portlandite was found, which was confirmed by the results of chemical composition analyses (point “1”—Figure 5b) and X-ray examinations (Figure 1).

In the case of cement paste 4 kept in both reaction environments, the SEM micrographs shown in Figure 2 indicate a clear change in the morphological structure, which is undoubtedly related to the corrosion process caused by the pig slurry. Cement paste subjected to biocorrosion is characterised by the presence of large, well-formed grains with a lamellar (plate) shape (Figure 2, sample 4_3). Moreover, in this sample, numerous pores (pitting) can be observed between the grain agglomerates. On the other hand, the paste kept in water is relatively compact and, in most cases, consists of isometric grains (Figure 2, sample 4_1).

**Figure 6:**
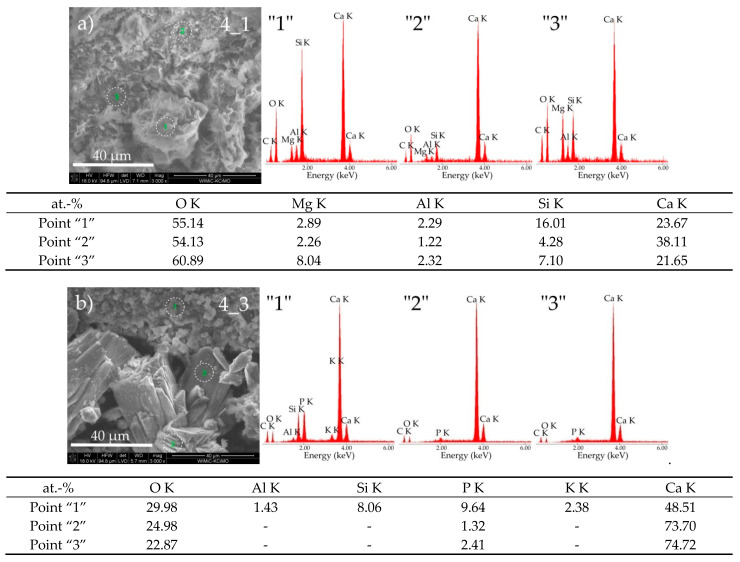
SEM micrographs of cement paste 4 obtained after exposure under the conditions of (**a**) water (sample 4_1) and (**b**) pig slurry (sample 4_3) with EDS quantitative point analyses from areas 1, 2, and 3.

Figure 6a,b show SEM micrographs of the cement paste exposed to both liquid media together with the results of EDS analyses of the chemical composition from selected areas of tested samples. In the lower part of the SEM photomicrograph (point “1”—Figure 6a), the C-S-H phase is visible, located presumably on the border of phase types II (mesh-like C-S-H phase II–honeycomb) and III according to the Diamond classification [16]; tricalcium aluminate (C_3_A) crystals are also visible, while in the area marked with point “3” (point “3”—Figure 6a), type III hydrated calcium silicates may dominate. In the upper part of the SEM photomicrograph (point “2”—Figure 6a), one can see fine portlandite plates fused with hydrated calcium silicates. In the cement paste exposed to pig slurry, large grains in the form of plates developed with a chemical composition similar to that of portlandite (points “2” and “3”—Figure 6b). Small spherical agglomerates visible in the upper part of the SEM photomicrograph (point “1”—Figure 6b) are probably a consequence of the process of calcium carbonate formation and phosphorus being incorporated into its unit cell, which can be observed in EDS spectra.

Noticeable differences in morphological structure were also observed in the case of cement paste 5, which was exposed to both of the discussed reaction environments.

**Figure 7:**
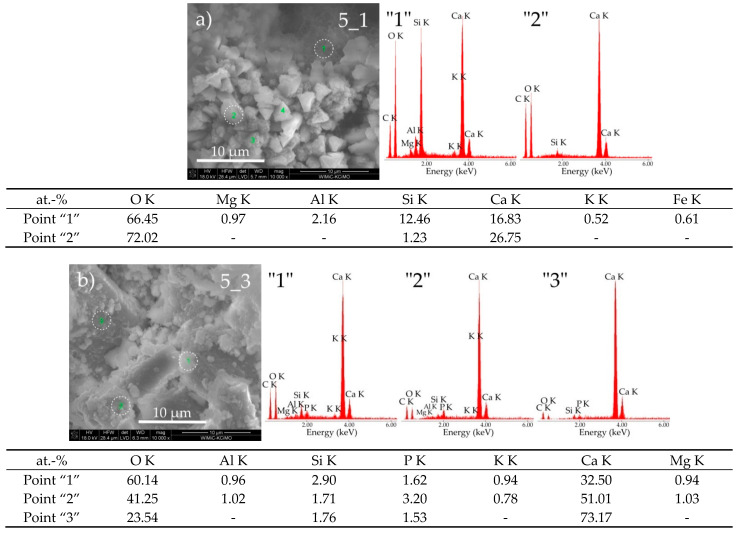
SEM micrographs of cement paste 5 obtained after exposure under the conditions of (**a**) water (sample 5_1) and (**b**) pig slurry (sample 5_3) with EDS quantitative point analyses from areas 1, 2, and 3.

As seen from Figure 2, cement paste 5 held in water (sample 5_1) is characterized by a compact structure consisting of irregular grains of size ranging from 5 to 40 µm. On the other hand, this sample, when subjected to biocorrosion, shows many fine grains, which usually grow into coarse crystalline grains with sizes from 5 to 30 µm (Figure 2, sample 5_3). In addition, numerous inter-agglomerate pores were observed in this sample. Figure 7a,b show SEM micrographs of both tested samples at a magnification of 10,000× and examples of EDS point out the spectra of chemical composition from selected areas of these samples. Sample 5_1 has a typical grout microstructure formed after hydration of Portland cement. In the vicinity of the porous C-S-H II phase (point “1”—Figure 7a), there are well-formed portlandite grains (points “2”, “3” and “4”—Figure 7a). Due to the chemical similarity of these grains, Figure 7a shows only the EDS point analysis spectrum from area “2”. In the case of sample 5_3, large, well-formed grains were observed, the chemical composition of which (points “1”, “2” and “3”—Figure 7b) approximately corresponds to portlandite grains, with phosphorus inclusions visible in EDS spectra.

**Figure 8:**
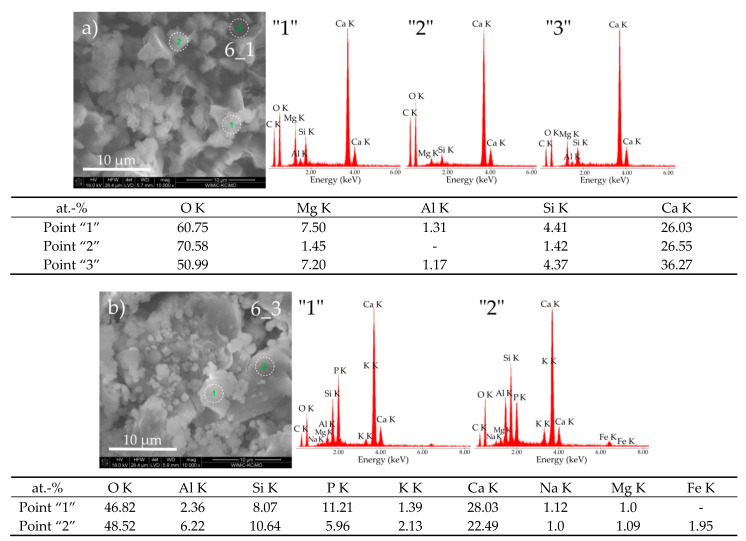
SEM micrographs of cement paste 6 obtained after exposure under the conditions of (**a**) water (sample 6_1) and (**b**) pig slurry (sample 6_3) with EDS quantitative point analyses from areas 1, 2, and 3.

From Figure 8, we can conclude that cement paste 6 had a reduced corrosion resistance in the environment of pig manure compared to the situation when the discussed material was kept in water.

After a six-week biocorrosion process, the cement sample showed the presence of well-formed grains with numerous pores or a network of channels with a large cross-section between them.

On the basis of the EDS point analysis of the chemical composition taken from the area marked point “1” (Figure 8a), one can conclude that in this part of the sample C-S-H I type phase with a fibrous structure, according to Diamond [16], exists. Additionally, precipitates in the form of C_3_A phase are visible. In the remaining areas of the sample (points “2” and “3”—Figure 8a), the presence of portlandite was identified.

**Figure 9:**
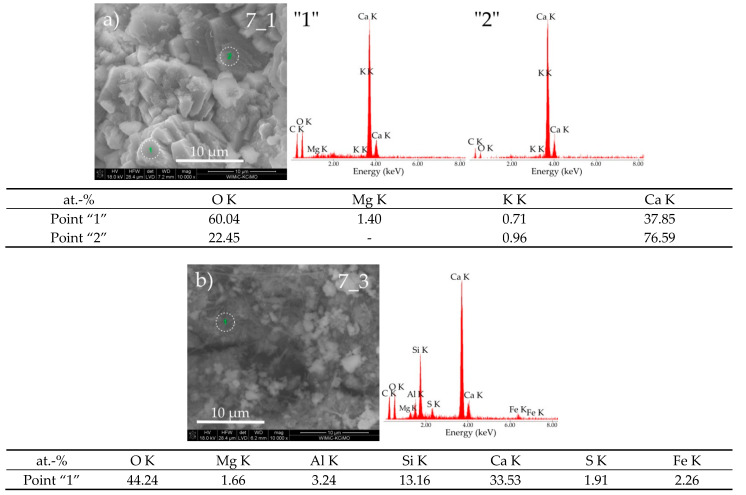
SEM micrographs of cement paste 7 obtained after exposure under the conditions of (**a**) water (sample 7_1) and (**b**) pig slurry (sample 7_3) with EDS quantitative point analyses from areas 1 and 2.

In sample 6_3, the presence of the C-S-H phase, presumably of type IV, was revealed as it resembles a compact amorphous gel (points “1” and “2”—Figure 8b). Along with this phase, the C_3_A phase probably coexists. In both of these phases, a high concentration of phosphorus was identified. Morphological observations of cement paste 7 also confirmed the significant influence of the corrosive medium on the deterioration process of this material. In the case of exposure of this cement paste in pig slurry, the appearance of numerous inter-agglomerate pores between well-formed large agglomerates consisting of fine grains (Figure 9b, sample 7_3) was found, while the cement paste sample kept in water was still compact and had a fine crystalline structure (Figure 9a, sample 7_1).

The SEM micrograph of sample 7_3 shows the presence of grains in the form of long needles corresponding to the crystals of taumasite, probably in the phase of transformation via the woodfordite solid solution of ettrigite and taumasite. This may be indicated by the presence of an increased content of carbon, aluminum, and silicon atoms indicated by EDS spectra, which occur in both discussed crystal phases (point “1”—Figure 9b). Well-formed crystallites with the given chemical composition (sample 7_3, points “1” and “2”—Figure 9a) correspond to the portlandite phase.

### 3.4. Biological and Biochemical Aspects of the Corrosion Process-Taumasite Formation Conditions

Taumasite (C_3_S∙CO_2_∙SO_3_∙15H_2_O) is a hydrated calcium sulphate–carbonate–silicate and one of the products of the sulphate corrosion of concrete, mortar, and cement pastes. Typically, taumasite belongs to the phases from the ettringite group. As a mineral, it occurs in the form of elongated, pointed crystal forms [17]. In concrete, mortar, or cement pastes, the occurrence of the taumasite phase is an undesirable phenomenon leading to the gradual destruction of the area occupied by hydrated calcium silicates (CSH). This in turn leads to a significant reduction of cohesion of these materials, an increase in porosity and, consequently, a decrease in mechanical strength [17,18,19].

Taumasite is most readily formed under strictly defined conditions such as the simultaneous presence of all the reactants and environmental conditions required for an effective reaction. In cement pastes, taumasite is formed under relatively low temperatures ranging from 0 to 15 °C [18,20] and assuming that the pH of the cement paste environment is lowered to about 10 [21]. The most intense dynamics of taumasite phase formation can be recorded at low temperatures between 0 and 3 °C.

Several mechanisms of the taumasite phase formation in the cement paste are described in the literature. The first one is the direct reaction of the C-S-H phase (hydrated calcium silicates) with calcium carbonate and calcium sulphate, which can be summarized as follows [20]:

3Ca^2+^ + SiO_3_^2−^ + CO_3_^2−^ + SO_4_^2−^ + 15H_2_O → 3CaO∙SiO_2_∙CO_2_∙SO_3_∙15H_2_O (1)

The second mechanism of taumasite formation is its formation by the so-called woodfordite, i.e., a solid solution of ettryngite and taumasite. The substrates in the above reaction are the C-S-H phase, the ettrigite formed earlier, as well as calcium carbonate, carbon dioxide and water [22,23,24]. The course of the reaction is shown below:

Ca_3_Si_2_O_7_⋅3H_2_O + Ca_6_[Al(OH)_6_]_2_(SO_4_)_3_⋅26H_2_O + CaCO_3_ + CO_2_ + xH_2_O
Ca_6_[Si(OH)_6_]_2_(CO_3_)_2_(SO_4_)_2_⋅24H_2_O + CaSO_4_⋅2H_2_O + Al_2_O_3_⋅xH_2_O + 3Ca(OH)_2_ (2)

In the present experiment, the conditions for the formation of taumasite are specific and more complex than those described in the examples above. Apart from the aggressive medium consisting of biological waste (slurry), the reaction of taumasite formation is probably accompanied by a number of other phenomena affecting the phase composition of the corrosive product. As previously mentioned, the formation of the taumasite phase requires the simultaneous presence of reaction ions, an appropriate pH and reduced ambient temperature. A very interesting phenomenon in this experiment is related to the synergistic effect of dead matter with living matter (bacteria), which has a direct impact on the formation of cement paste corrosion. The bacteria present in the pig slurry solution release carbon dioxide in the process of respiration, which in turn reacts with the crystallizing calcium hydroxide phases resulting from the cement hydration reaction, leading to the formation of highly active calcium carbonate. Moreover, the pH of the corrosive medium neutralizes the reaction of the cement paste, creating almost ideal conditions for the corrosion process. It is of importance that calcium carbonate crystallizing as a result of the earlier discussed mechanism is doped with a significant amount of phosphorus and other ions present in the slurry, which certainly affects its reactivity during the formation of taumasite during the corrosion of pastes.

The concept developed in the above experiment needs further investigation concerning the aspect of calcium carbonate formation in the reaction with living matter (bacteria). The influence of slurry and the rate of the formation of corrosive phases as a result of the exposure of cement pastes to slurry solutions is also not yet fully understood.

## 4. Conclusions

The subject of this work was the biological corrosion of seven types of Portland cements produced by cement plants located in Poland. The tested cements were characterized by a similar strength class (amounting to 42.5) and they were of a similar type (CEM I). They contained 95 to 100% clinker and 0 to 5% of the secondary component in the form of CaSO_4_∙2H_2_O dihydrate gypsum as a regulator of the setting time. The reason for testing this type of cement was the high concentration of calcium oxide, silicon dioxide, and aluminum oxide.

The presence of the abovementioned oxides is related to the tricalcium silicate and tricalcium aluminate as a dominant phase. The aforementioned phases undergoing the process of hydration favor the formation of large amounts of CSH, calcium hydroxide, and ettringite. The indicated products are characterized by a high susceptibility to biocorrosion in an aggressive reaction environment such as pig slurry.

The cement pastes were kept under a corrosive environment for a period of six weeks. In the same period of time, respective samples were stored in water as the reference medium. Based on the results of analyses of the phase composition of cement pastes obtained after exposure to pig slurry conditions, the existence of the corrosion product in the form of taumasite [C_3_S∙CO_2_∙SO_3_∙15H_2_O] was confirmed in four cement pastes marked as 1_3, 2_3, 3_3, and 7_3.

The disappearance of tricalcium aluminate in these cement pastes as a result of total hydration leading to the formation of corrosion products is noteworthy. Apart from portlandite (Ca(OH)_2_), significant amounts of calcium carbonate, probably formed as a result of a topochemical reaction involving active calcium hydroxide with carbon dioxide released by bacteria in pig slurry, were identified in the phase compositions of the cement pastes. The presence of calcium carbonate within the corrosion products results from the formation of taumasite during the biological degradation of the tested cement pastes. In addition, small amounts of unreacted dicalcium silicate are also visible in the tested samples subjected to the corrosion process. In turn, in samples 4_3, 5_3, and 6_3 not fully reacted tricalcium aluminate was present. Moreover, calcium carbonate, portlandite, and dicalcium silicate were found. The taumasite phase was not identified in these samples.

In all the analyzed materials subjected to the corrosion process in pig slurry, a slight increase in the background on the X-ray diffraction pattern in the range of 25 to 40 2θ was observed, which confirms the presence of submicrocrystalline C-S-H phases. The microscopic observations supported by EDS analysis of the chemical composition of the tested samples confirmed the presence of most of the phases detected by XRD. Hydrated calcium silicates of CSH type with different morphology according to the Diamond classification were observed.

Crystallites of calcium carbonate and portlandite were found in all the materials. Semi-quantitative EDS analysis of the chemical composition of corrosion products confirmed a significant concentration of phosphorus originating from pig slurry. The presented research results constitute a prerequisite for further research aimed at explaining the complex mechanism of the biological corrosion of both mortar and concrete prepared on the basis of selected Portland cements and special cements that can be used in the production of innovative building materials for the agricultural industry.

## Figures and Tables

**Table 1:** Chemical composition of OCP (ordinary Portland cement).

Oxides/ Ions [%]	Chełm CEM I 42.5N	Rudniki CEM I 42.5R	Górażdze CEM I 42.5R	Ożarów CEM I 42.5N	Odra CEM I 42.5R	Warta CEM I 42.5R	Małogoszcz CEM I 42.5R
	1_3	2_3	3_3	4_3	5_3	6_3	7_3
SiO_2_	21.70	20.81	21.66	21.56	19.36	22.47	24.27
Al_2_O_3_	3.30	4.69	5.14	4.76	5.75	6.05	4.29
Fe_2_O_3_	4.56	3.77	2.77	3.14	2.83	2.72	2.80
CaO	65.52	65.79	65.36	64.94	66.99	63.67	64.01
MgO	1.12	1.33	1.46	1.92	1.68	1.91	1.10
SO_3_	3.14	2.65	2.52	2.63	2.20	2.12	2.30
K_2_O	0.41	0.77	0.86	0.71	1.05	0.80	1.10
Na_2_O	0.20	0.14	0.15	0.30	0.11	0.20	0.10
Cl^−^	0.05	0.05	0.08	0.04	0.03	0.06	0.03
Total	100.0	100.0	100.0	100.0	100.0	100.0	100.0

**Table 2:** Chemical composition of water used in the experiments.

Parameter	Unit	Value
Arsen	mg/L	<0.001
Nitrates	mg/L	1.60 ± 0.03
Cyanides	mg/L	<0.005
Fluorides	mg/L	0.41 ± 0.01
Magnesium	mg/L	14.00 ± 0.11
Copper	mg/L	<0.003
Lead	mg/L	<0.001
Mercury	mg/L	<0.0001
Sulfate	mg/L	175.0 ± 12.1
Total hardness CaCO3	mg/L	377.0 ± 18.6
Calcium	mg/L	87.0 ± 1.9
Iron	mg/L	0.20 ± 0.01
Total trihalomethanes (THM)	µg/L	3.0 ± 0.5
Total chlorates and chlorites	mg/L	0.100 ± 0.006

**Table 3:** Chemical composition of liquid pig slurry.

Parameter	Unit	Value
Kjeldahl total nitrogen	mg/L	1240.0 ± 60.4
Ammonium nitrogen	mg/L	1050.0 ± 51.1
Total nitrogen	mg/L	1350.0 ± 63.5
Nitrite nitrogen	mg/L	0.032 ± 0.003
Nitrate nitrogen	mg/L	0.31 ± 0.05
Chrome	mg/L	0.40 (-) *
Cadmium	mg/L	0.05 ± 0.01
Nickel	mg/L	0.09 ± 0.01
Lead	mg/L	<0.5
Mercury	mg/L	<0.003
Calcium	mg/L	68 (-) *
Magnesium	mg/L	4.37 ± 0.87
Total phosphorous	mg/L	352 (-)
Potassium	mg/L	684.0 ± 15.4
Dry mass	mg/L	1.31 ± 0.06

* single measurement.

**Table 4:** Weight of samples made of cement pastes (g).

Sample	I–Initial Mass	Uncertainty of the Result [±]	II–Exposure Time Two Weeks	Uncertainty of the Result [±]	III–Exposure Time Four Weeks	Uncertainty of the Result [±]	IV–Exposure Time Six Weeks	Uncertainty of the Result [±]
Chełm (1)	136	0.26	142	0.27	138	0.26	137	0.26
Rudniki (2)	139	0.26	139	0.26	138	0.26	140	0.26
Górażdże (3)	139	0.26	133	0.25	136	0.26	138	0.26
Ożarów (4)	138	0.26	136	0.26	135	0.25	138	0.26
Odra (5)	143	0.27	143	0.27	141	0.27	141	0.27
Warta(6)	136	0.26	139	0.26	135	0.25	134	0.25
Małogoszcz (7)	137	0.26	136	0.26	135	0.25	135	0.25

**Table 5:** Phase composition of cement pastes [“+”—phase present in the sample; “−”—phase not detected in the sample].

Sample	Phase Composition/Abbreviation/ICDD *				
	Ca(OH)_2_ /P/ [4–773] *	CaCO_3_/K/ [24–27] *	C_2_S/L/ [33–302] *	C_3_A/G/ [38–1429] *	C_3_S·CO2·SO_3_·15H_2_O/T/[46–1360] *
1_3	+	+	+	−	+
2_3	+	+	+	−	+
3_3	+	+	+	−	+
4_3	+	+	+	+	−
5_3	+	+	+	+	−
6_3	+	+	+	+	−
7_3	+	+	+	−	+

* ICDD—International Centre for Diffraction Data record.

## Data Availability

Data available on request.
